# Letter to the editor: testing the generalizability of DeepPlantAllergy on challenging allergen prediction scenarios

**DOI:** 10.1093/bib/bbag149

**Published:** 2026-04-06

**Authors:** Kazim Okan Dolu

**Affiliations:** Department of Pediatric Allergy and Immunology, Kanuni Sultan Süleyman Training and Research Hospital, Atakent Mah., Turgut Özal Bulvarı No. 1, Küçükçekmece, 34303, Istanbul, Turkey

**Keywords:** allergenicity prediction, deep learning, evaluation leakage, hard negatives, class imbalance, AUPRC

## Abstract

Dhouib *et al*. (DeepPlantAllergy: deep learning for explainable prediction of allergenicity in plant proteins. *Brief Bioinform* 2025;**26**:bbaf605.) developed DeepPlantAllergy, a deep learning model for predicting allergenicity in plant proteins, reporting area under the receiver operating characteristic curve (ROC-AUC) ≈ 97.7–97.8% on an independent test set. However, the dataset construction may lead to optimistic performance estimates. Specifically, non-allergen sequences sharing >20% identity with allergens were removed before the train/test split, which can reduce the presence of “hard negatives” (moderately similar non-allergens) in the test set and thereby weaken assessment under realistic screening conditions. Because practical allergen screening requires discrimination against large numbers of non-allergens that may share moderate sequence identity, we suggest re-evaluating the model using test sets that retain challenging negatives (with filtering performed against training allergens only) and reporting precision–recall metrics (area under the precision–recall curve) alongside ROC-AUC to better reflect performance under class imbalance.

I read the article from Dhouib *et al*. with interest, as they developed a deep learning model, DeepPlantAllergy, to predict allergenicity in plant proteins [1]. The model reported exceptional performance with area under the receiver operating characteristic curve (ROC-AUC)≈ 97.7%–97.8% on the independent test set (*N* = 121; one sequence filtered due to nonstandard amino acids) [1]. However, I have concerns about certain aspects of the dataset construction that may affect the interpretation of these performance metrics and limit real-world applicability.

The “Data preparation” section describes removing non-allergen sequences sharing >20% identity with allergens ‘prior to the train/test split’ [1]. While the motivation for reducing similarity-based bias is understandable, this approach may inadvertently introduce evaluation leakage. By filtering negatives against ‘all’ allergens—including those later assigned to the test set—the procedure effectively sanitizes the test set by removing “hard negatives” (non-allergenic proteins with moderate sequence similarity to allergens).

In real-world allergen screening scenarios, models must distinguish allergens from thousands of non-allergens that may share 20%–50% sequence identity. Given that allergens are distributed into a limited number of protein families [2], the ability to distinguish allergens from moderately similar non-allergens ‘within and across these families’ becomes particularly important for practical screening applications. By preemptively eliminating such cases from the test set, the classification task becomes less representative of practical challenges.

This concern is particularly relevant given that the authors state they “lowered the identity threshold to 20% to enable the assessment of advanced embeddings... in capturing similarities below the twilight zone” [1]. However, to fully assess this capability, the test set would ideally ‘retain’ non-allergens with moderate similarity to allergens. The current approach removes precisely the cases needed to validate the model’s discriminative power in this challenging similarity range.

Additionally, realistic allergen screening involves extreme class imbalance. Even at 95% specificity, a model screening 10 000 proteins (assuming 10 allergens detected with high sensitivity) would yield ~500 false positives alongside 10 true positives, resulting in a positive predictive value <2%. The limited number of positive test cases (*n* = 61) may also affect the precision of performance estimates; with 61 positives, one additional error shifts positive-class recall by ~1.6 percentage points.

To better understand DeepPlantAllergy’s performance in practical scenarios, I suggest the authors consider re-evaluating the model on: (1) a test set that includes non-allergens with 20%–50% sequence identity to known allergens, with negative filtering performed against training allergens only; and (2) the full negative set without downsampling, since the current evaluation uses a 1:1 allergen-to-non-allergen ratio obtained by downsampling negatives [1], to assess performance under realistic class imbalance, reporting AUPRC (area under the precision–recall curve) in addition to ROC-AUC, as AUPRC is more informative for highly imbalanced datasets [3]. Such validation would provide valuable insights into the model’s utility for practical allergen screening applications.

**Key Points**

- Presplit negative filtering may remove hard negatives and yield optimistic test performance.
- A 1:1 class ratio can overstate practical utility under extreme class imbalance.
- External validation with challenging negatives and imbalanced prevalence would strengthen applicability.

## Data Availability

No new data were generated or analyzed in this study.
